# Supplementary material for: Interferon-γ signaling synergizes with LRRK2 in neurons and microglia derived from human induced pluripotent stem cells
Source: Nat Commun. 2020 Oct 14;11:5163. doi: 10.1038/s41467-020-18755-4 (PMC7560616; doi:10.1038/s41467-020-18755-4)
Supplement: Supplementary file 1 — Supplementary Information [file 41467_2020_18755_MOESM1_ESM.pdf]

SUPPLEMENTARY INFORMATION

Interferon-γ signaling synergizes with LRRK2 in neurons and microglia derived from human induced pluripotent stem cells

Vasiliki Panagiotakopoulou, Dina Ivanyuk, Silvia De Cicco, Wadood Haq, Aleksandra Arsić, Cong Yu, Daria Messelodi, Marvin Oldrati, David Schöndorf, Maria-Jose Perez, Ruggiero Pio Cassatella, Meike Jakobi, Nicole Schneiderhan-Marra, Thomas Gasser, Ivana Nikić-Spiegel, Michela Deleidi*

*Manuscript correspondence:
Michela Deleidi; michela.deleidi@dzne.de

Supplementary Methods

Primers used for qRT-PCR

RPLP0	FW	CCTCATATCCGGGGGAATGTG
	RV	GCAGCAGCTGGCACCTTATTG
HMBS	FW	ATGCCCTGGAGAAGAATGAAGT
	RV	TTGGGTGAAAGACAACAGCATC
β2-microglobulin	FW	GATAGTTAAGTGGGATCGAG
	RV	GCAAGCAAGCAGAATTTGGA
LRRK2	FW	TCCCTGCCATACGAGATTACC
	RV	GCACATTTTTACGCTCCGATA
NFAT1	FW	AGAAACTCGGCTCCAGAATCC
	RV	TGGTTGCCCTCATGTTGTTTTT
NFAT2	FW	GCCGCAGCACCCCTACCAGT
	RV	TTCTTCCTCCCGATGTCCGTCTCT
NFAT3	FW	TCAGAAGACACGGCGGACTTCC
	RV	TGAACATCTGTAGGGTCAGTGG
NFAT4	FW	ACCAGCCCGGGAGACTTCAATAGA
	RV	AAATACCTGCACAATCAATACTGG
PLGC2	FW	TCCACCACGGTCAATGTAGAT
	RV	~~C~~CCCTGGGCGGATTTCTTTTAT
IFNGR1	FW	CATCACGTCATACCAGCCATTT
	RV	CTGGATTGTCTTCGGTATGCAT
SRC	FW	TGGCAAGATCACCAGACGG
	RV	GGCACCTTTCGTGGTCTCAC
PIK3CB	FW	CTAATGTGTCAAGTCGAGGTGGAA
	RV	GGAAAATCTCTCGGCAGTCTTGT
IFNG	FW	TGACCAGAGCATCCAAAAGA
	RV	CTCTTCGACCTCGAAACAGC
MAPK14	FW	TTCTGTTGATCCCACTTCACTGT
	RV	ACACACATGCACACACACTAAC
AKT1	FW	ATGAGCGACGTGGCTATTGTGAAG
	RV	GAGGCCGTCAGCCACAGTCTGGATG
EP300	FW	AAACCCACCAGATGAGGAC
	RV	TATGCACTAGATGGCTCCGCAG
AKT2	FW	ATGAATGAGGTGTCTGTCATCAAAGAAGGC
	RV	TGCTTGAGGCTGTTGGCGACC
CAMK2G	FW	ACCCGTTTCACCGACGACTA
	RV	CTCCTGCGTGGAGGTTTTCTT
PIK3CD	FW	CAACCAGACAGCGGAGCAGCAAGA
	RV	CACTTCTGGGTCGCACAAGGAGTCAA
PIK3R1	FW	TGACGCTTTCAAACGCTATC
	RV	CAGAGAGTACTCTTGCATTC
MAPK1	FW	CCAGAGAACCCTGAGGGAGA
	RV	TGTTGAGCAGCAGGTTGGAA
IFNGR2	FW	GCTGCTCGGAGTCTTCGCCG
	RV	GGACTGGCGGCAGTGAAGTC
CBLB	FW	CGGTAATTGTTGCGTTTCCA
	RV	ACAGCTCGCTCCCGAAGAA

CDKN1A	FW	GGCAGACCAGCATGACAGATT
	RV	GCGGATTAGGGCTTCCTCT
MAP2K6	FW	GGTTGTCTGGTCACTGAGGTCAC
	RV	CAGGGAACCTGAGACAGGCTAC
MYC	FW	GGCCCCCAAGGTAGTTATCCTT
	RV	CGTTTCCGCAACAAGTCCTCT
PRKCD	FW	CCCTTCTGTGCCGTGAAGAT
	RV	GCCCGCATTAGCACAATCTG
MAPK3	FW	CTGGATCAGCTCAACCACATT
	RV	AGAGACTGTAGGTAGTTTCGGG
CREBBP	FW	CAACCCCAAAAGAGCCAAACT
	RV	CCTCGTAGAAGCTCCGACAGT
SMAD7	FW	TACCGTGCAGATCAGCTTTG
	RV	TTTGCATGAAAAGCAAGCAC
PTPN11	FW	GTGGAGGAGAACGGTTTGATTC
	RV	CCAATGTTTCCACCATAGGATTC
BRCA1	FW	GGCTATCCTCTCAGAGTGACATTT
	RV	GCTTTATCAGGTTATGTTGCATGGT
CEBPB	FW	CACAGCGACGACTGCAAGATCC
	RV	CTTGAACAAGTTCCGCAGGGTG
JAK2	FW	GATAAAGCACACAGAAACTATTCAGAGTC
	RV	AGAATATTCTCGTCTCCACAAAC
eIF2AK2	FW	TCTTCATGTATGTGACACTGC
	RV	CACACAGTCAAGGTCCTTAG
AKT3	FW	ATGAGCGATGTTACCATTGT
	RV	CAGTCTGTCTGCTACAGCCTGGATA
ICAM1	FW	AGGCCACCCCAGAGGACAAC
	RV	CCCATTATGACTGCGGCTGCTA
SOCS1	FW	TGGTAGCACACAACCAGGTG
	RV	GAACGGAATGTGCGGAAGT
IRF1	FW	TGCATTTATTTATACAGTGCCTTGCT
	RV	CCCTCCCTGGGCCTGTTGAG
STAT1	FW	CTAGTGGAGTGGAAGCGGAG
	RV	CACCACAAACGAGCTCTGAA

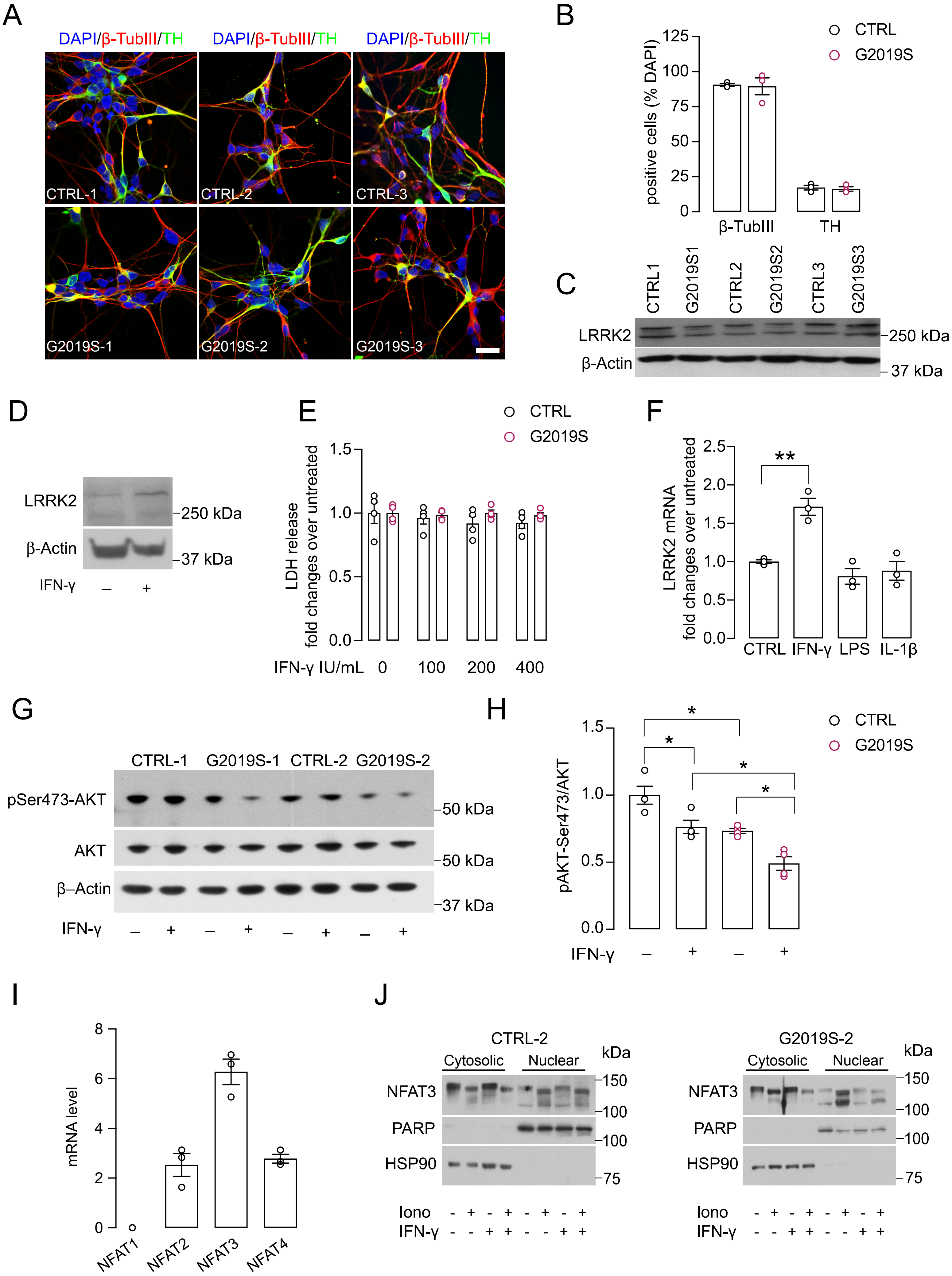

A
DAPI/β-TubIII/TH
CTRL-1
CTRL-2
CTRL-3
G2019S-1
G2019S-2
G2019S-3
B
positive cells (% DAPI)
CTRL
G2019S
β-TubIII
TH
C
CTRL1
G2019S1
CTRL2
G2019S2
CTRL3
G2019S3
LRRK2
β-Actin
250 kDa
37 kDa
D
LRRK2
β-Actin
250 kDa
37 kDa
IFN-γ
E
LDH release
fold changes over untreated
IFN-γ IU/mL
F
LRRK2 mRNA
fold changes over untreated
CTRL
IFN-γ
LPS
IL-1β
G
CTRL-1
G2019S-1
CTRL-2
G2019S-2
pSer473-AKT
AKT
β–Actin
50 kDa
37 kDa
IFN-γ
H
pAKT-Ser473/AKT
I
mRNA level
NFAT1
NFAT2
NFAT3
NFAT4
J
CTRL-2
G2019S-2
Cytosolic
Nuclear
kDa
NFAT3
PARP
HSP90
Iono
IFN-γ

Supplementary Figure 1. IFN-γ synergizes with *LRRK2* G2019S in the negative regulation of NFAT3 shuttling in human neurons. (A) Immunostaining of differentiated NPC-derived neuronal cultures from *LRRK2* G2019S iPSCs and corresponding isogenic controls. Cells were stained for TH (green) and β-TubIII (red) and nuclei were counterstained with DAPI (blue). Scale bar, 20 μm. **(B)** Quantification of the percentage of β-TubIII- and TH-positive neurons (mean ± SEM, n=3 independent experiments). **(C)** Representative Western blot of LRRK2 levels in neuronal cultures from *LRRK2* G2019S iPSCs and corresponding isogenic controls. **(D)** iPSC-derived cortical neurons were treated with 200IU/mL IFN-γ for 24 hrs or left untreated; representative Western blot of LRRK2 levels is shown. **(E)** *LRRK2* G2019S neurons and controls were treated with IFN-γ at given concentrations and cytotoxicity was evaluated by LDH assay. LDH release values were calculated as a percentage relative to control untreated cells lysed with Triton X-100 (mean ± SEM; n=4 independent experiments). **(F)** *LRRK2* mRNA levels in control iPSC-derived neurons treated with 200 IU/mL IFN-γ, 100 ng/mL LPS, or 100 IU/mL IL-1β for 24 hrs (mean ± SEM, one-way ANOVA, Bonferroni post hoc, ***P*=0.0025; n=3 independent experiments). **(G)** Representative Western blot of p-AKT Ser473 and total AKT in *LRRK2* G2019S neurons (G2019S-1, G2019S-2) and corresponding isogenic controls (CTRL-1, CTRL-2). Treatment with 200 IU/mL IFN-γ is indicated. **(H)** Quantification of pAKT/AKT levels in *LRRK2* G2019S neurons and isogenic controls. Data are normalized to CTRL (mean ± SEM, two-way ANOVA, Bonferroni post hoc, **P*=0.0315, 0.0145, 0.0264, 0.0122 in sequence; n=4 independent experiments). **(I)** mRNA expression of NFAT isoforms in control iPSC-derived neurons measured by qRT-PCR (mean ± SEM; n=3 independent experiments). **(J)** Representative Western blots of NFAT3 in nuclear and cytosolic fractions of control (left panel) and isogenic *LRRK2* G2019S neurons (right panel). Treatment with 1 μM ionomycin for 30 min and/or 200 IU/mL IFN-γ for 24 hrs is indicated.

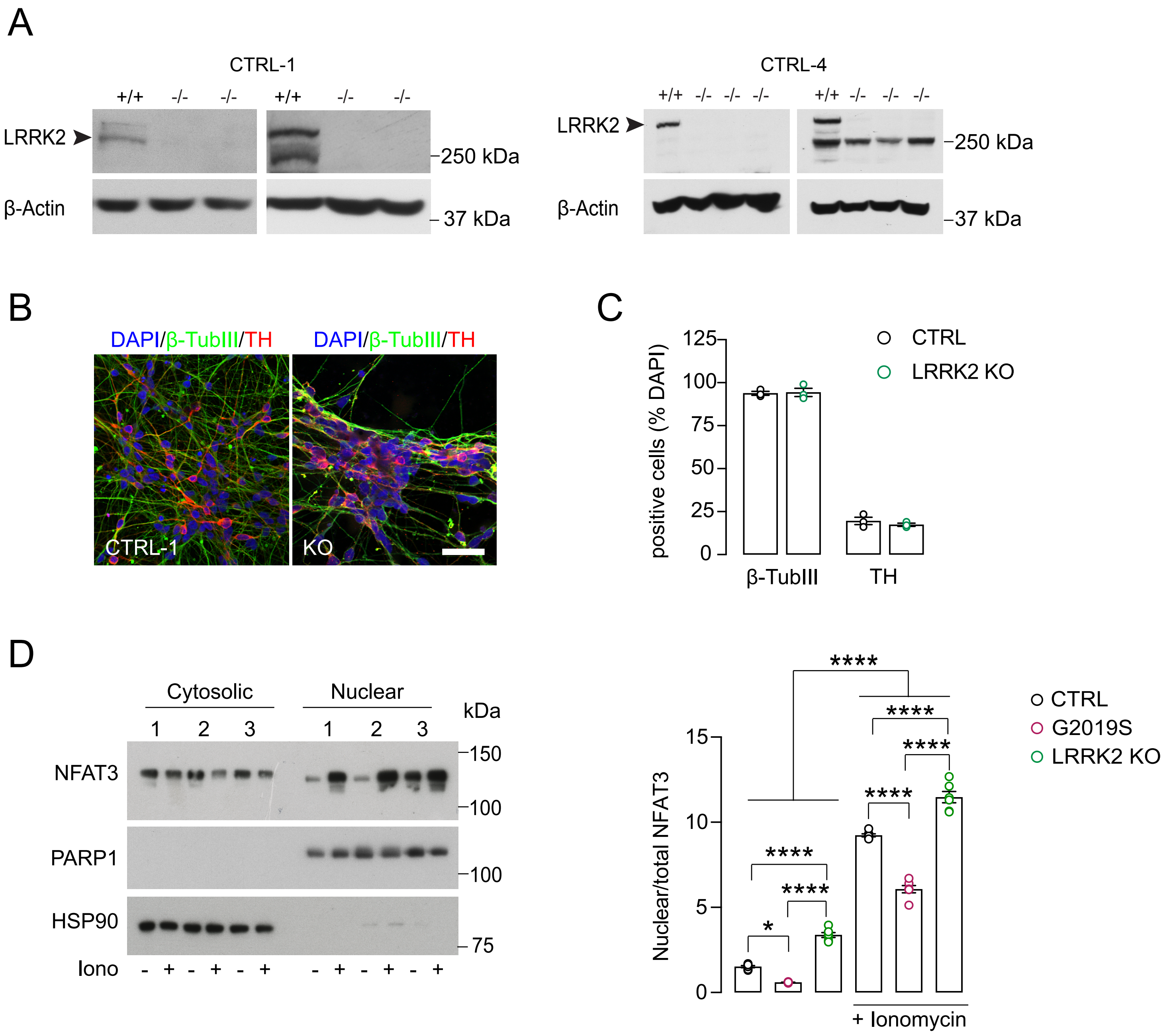
A
CTRL-1
+/+ -/- -/- +/+ -/- -/-
LRRK2
β-Actin
250 kDa
37 kDa
CTRL-4
+/+ -/- -/- -/- +/+ -/- -/- -/-
LRRK2
β-Actin
250 kDa
37 kDa
B
DAPI/β-TubIII/TH
DAPI/β-TubIII/TH
CTRL-1
KO
C
positive cells (% DAPI)
125
100
75
50
25
0
CTRL
LRRK2 KO
β-TubIII
TH
D
Cytosolic
Nuclear
1 2 3
1 2 3
kDa
NFAT3
PARP1
HSP90
150
100
100
75
Iono
- + - + - + - + - + - +
Nuclear/total NFAT3
15
10
5
0
****
****
****
****
****
****
*
+ Ionomycin
CTRL
G2019S
LRRK2 KO

Supplementary Figure 2. Generation of *LRRK2* KO human iPSCs. (A) *LRRK2* KO iPSCs (CTRL1) were generated using ZFNs and a targeting vector harboring a premature stop codon in *LRRK2* exon 41 (left). LRRK2 KO iPSCs (CTRL 4) were generated using CRISPR-Cas9. Two single-guide RNAs (sgRNAs) targeting exon 3 of *LRRK2* were used, leading to a deletion that results in an early stop codon (right). Representative Western blot for LRRK2, showing the absence of LRRK2 in human iPSC-derived microglia. The following antibodies were used: UDD3 30(12) and 24D8 (left panel), 1E11 and 24D8 (right panel). **(B)** Immunostaining of differentiated iPSC-derived neuronal cultures from *LRRK2* KO and isogenic controls. Cells were stained for TH (red) and β-TubIII (green) and nuclei were counterstained with DAPI (blue). Scale bar, 20 µm. **(C)** Quantification of the percentage of β-TubIII-positive and TH-positive neurons (mean ± SEM, n=3 independent experiments). **(D)** Representative Western blot of NFAT3 in nuclear and cytosolic fractions of control (1), *LRRK2* G2019S (2) and *LRRK2* KO (3) neurons. Treatment with 1 µM ionomycin for 30 min is indicated. On the right, quantification of nuclear to total (nuclear and cytosolic fraction) NFAT3 (mean ± SEM, two-way ANOVA, Bonferroni post hoc, *****P*<0.0001, **P*=0.0150; n=6 independent experiments).

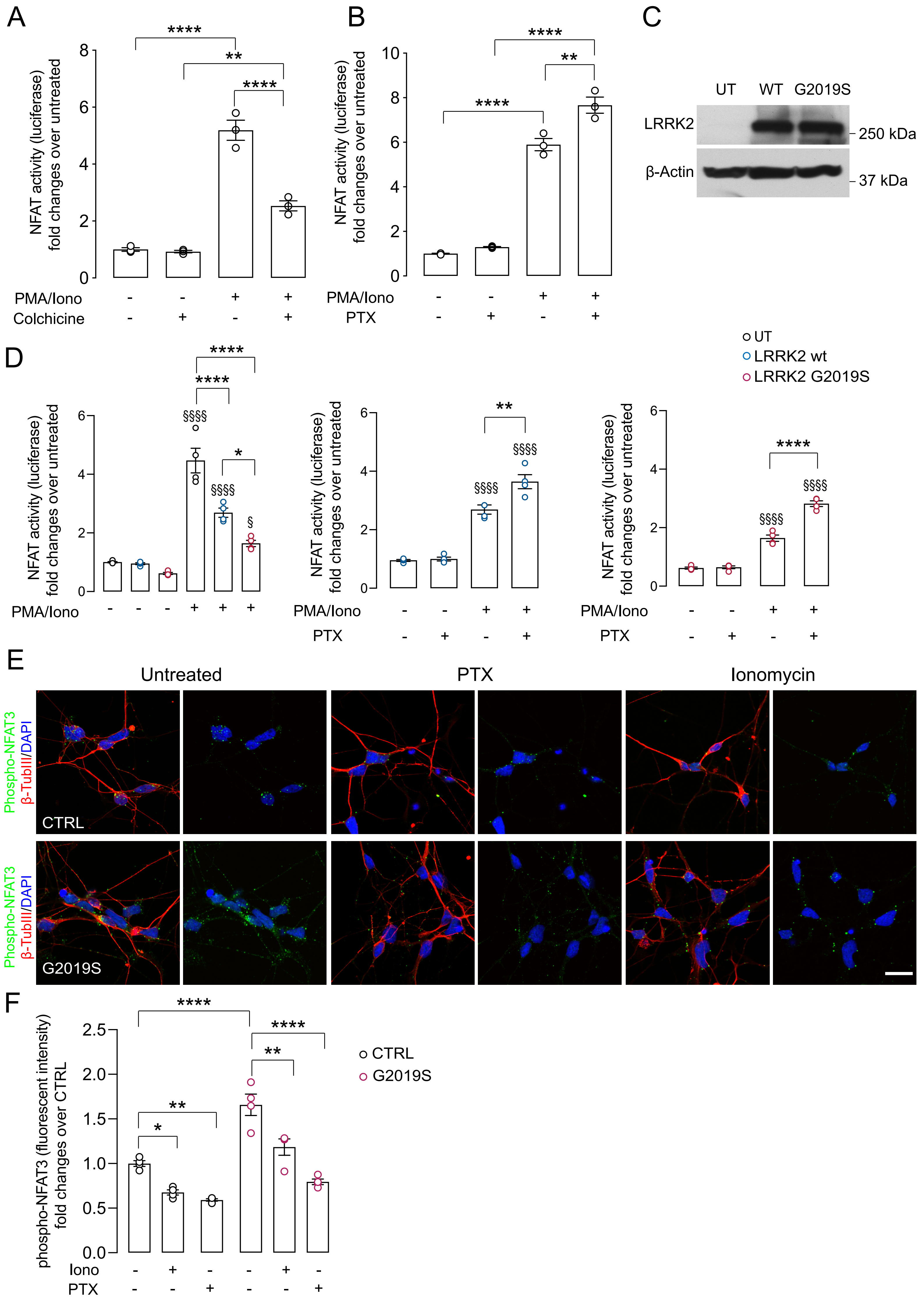

A
NFAT activity (luciferase) fold changes over untreated
PMA/Iono
Colchicine
B
PTX
C
UT
WT
G2019S
LRRK2
β-Actin
250 kDa
37 kDa
D
UT
LRRK2 wt
LRRK2 G2019S
E
Untreated
PTX
Ionomycin
Phospho-NFAT3
β-TubIII/DAPI
CTRL
G2019S
F
phospho-NFAT3 (fluorescent intensity) fold changes over CTRL
CTRL
G2019S
Iono
PTX

Supplementary Figure 3. Microtubule network regulates NFAT shuttling. HEK293 reporter cell lines expressing the luciferase gene under a promoter with NFAT-response elements were treated with microtubule-stabilizing (Paclitaxel, PTX) and destabilizing (Colchicine) agents. **(A)** NFAT-driven luciferase expression in NFAT Reporter - HEK293 cells treated with vehicle or 10μM colchicine for 30 min and stimulated with PMA/Ionomycin (mean ± SEM, one-way ANOVA, Bonferroni post hoc, ****P<0.0001, **P=0.0029; n=3 independent experiments). **(B)** NFAT-driven luciferase expression in NFAT Reporter - HEK293 cells treated with vehicle or 100 nM PTX for 24 hrs and stimulated with PMA/Ionomycin (mean ± SEM, one-way ANOVA, Bonferroni post hoc, ****P<0.0001; **P=0.0035; n=3 independent experiments). **(C)** Western blot for LRRK2 levels in HEK293 cells untransfected (UT) or transfected with wt or LRRK2 G2019S. **(D)** NFAT-driven luciferase expression in NFAT Reporter - HEK293 cells overexpressing wt or G2019S LRRK2 and stimulated with PMA/Ionomycin alone (left) or in combination with 100 nM PTX treatment (middle and right panels) for 24 hrs. Data are expressed as fold changes over UT untreated (mean ± SEM, one-way and two-way ANOVA, Bonferroni post hoc, §§§§P<0.0001, relative to corresponding untreated; § P=0.0187; ****P<0.0001, **P=0.0040; *P=0.0155, n=4 independent experiments). **(E)** Representative confocal images of phospho-NFAT3 immunostaining (green) in *LRRK2* G2019S and isogenic control iPSC-derived neurons treated with 100 nm PTX for 24 hrs or 1 μM ionomycin for 30 min. β-TubIII (red), DAPI staining (blue). Scale bar, 20 μm. **(F)** Quantification of phospho-NFAT3 fluorescent intensity as assessed by Image J ROI analysis (mean ± SEM, two-way ANOVA, Bonferroni post hoc, ****P<0.0001, **P=0.0049, 0.0011 in sequence; *P=0.0368, n=4 independent experiments).

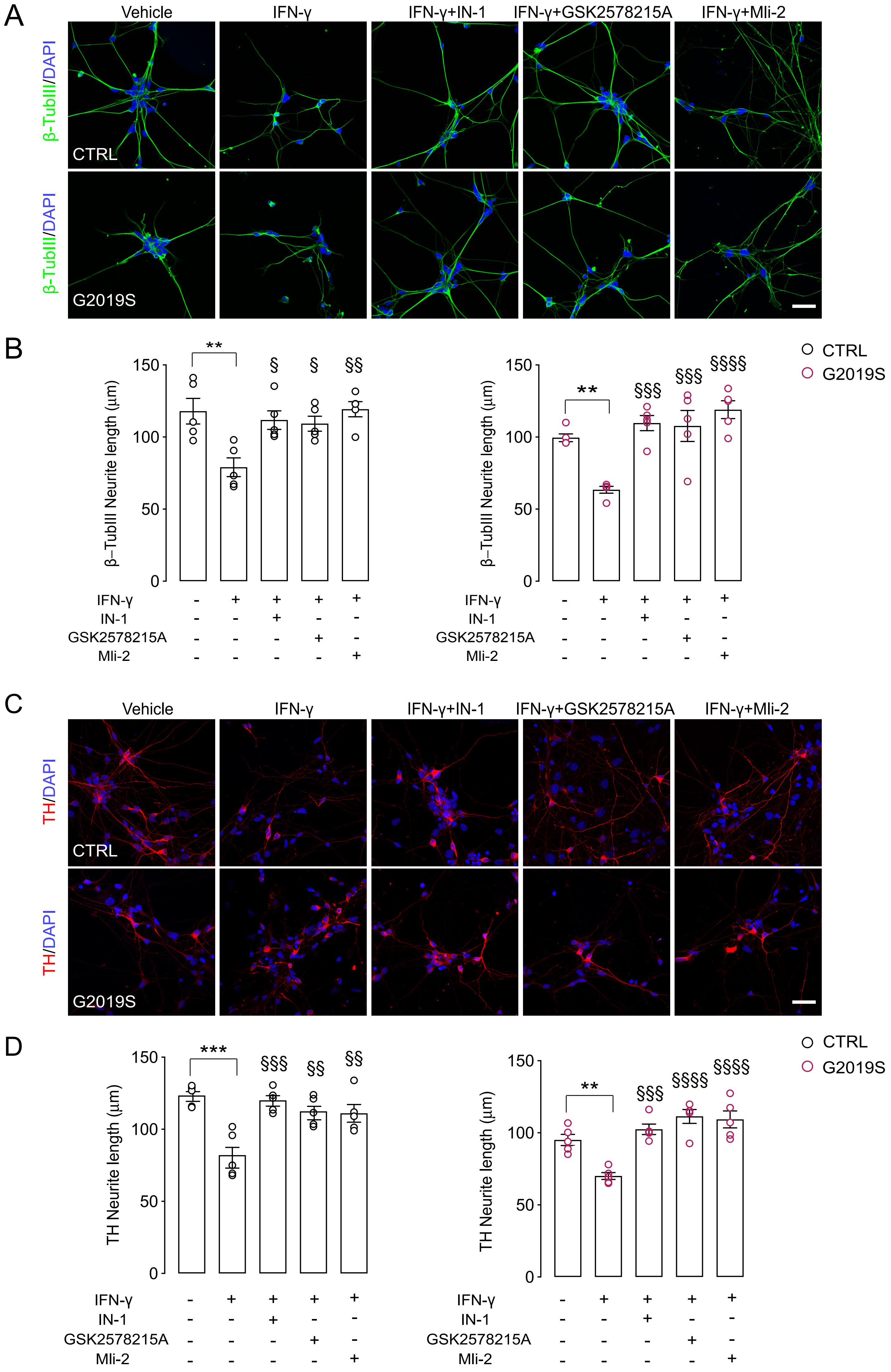
A
Vehicle
IFN-γ
IFN-γ+IN-1
IFN-γ+GSK2578215A
IFN-γ+Mli-2
β-TubIII/DAPI
CTRL
β-TubIII/DAPI
G2019S
B
β−TubIII Neurite length (μm)
150
100
50
0
**
§
§
§§
**
§§§
§§§
§§§§
CTRL
G2019S
IFN-γ - + + + +
IN-1 - - + - -
GSK2578215A - - - + -
Mli-2 - - - - +
C
Vehicle
IFN-γ
IFN-γ+IN-1
IFN-γ+GSK2578215A
IFN-γ+Mli-2
TH/DAPI
CTRL
TH/DAPI
G2019S
D
TH Neurite length (μm)
150
100
50
0
***
§§§
§§
§§
**
§§§
§§§§
§§§§
CTRL
G2019S
IFN-γ - + + + +
IN-1 - - + - -
GSK2578215A - - - + -
Mli-2 - - - - +

Supplementary Figure 4. IFN-γ reduces neurite outgrowth by decreasing NFAT activity. *LRRK2* G2019S and isogenic control iPSC-derived neurons were treated with 200IU/mL IFN-γ for 24 hrs with or without prior LRRK2 kinase inhibition with 1,5μM IN-1 or 2 μM GSK2578215A or 100nM Mli-2. **(A)** Representative images of β-TubIII immunostaining (green) showing neurite elongation; DAPI staining (blue). Scale bar, 50 μm. **(B)** Quantification of β-TubIII-positive neurite elongation in control (left panel) and *LRRK2* G2019S (right panel) iPSC-derived neurons (mean ± SEM, one-way ANOVA, Bonferroni post hoc, IFN-γ-treated vs untreated ***P*= 0.0046, 0.0055 in sequence; LRRK2 kinase inhibitor-treated vs untreated §§§§*P*<0.0001, §§§*P*=0.0004, 0.0006 in sequence, §§*P*=0.0032, §*P*=0.0217, 0.0401 in sequence; n=5 independent experiments). **(C)** Representative images of TH immunostaining (red) showing neurite elongation; DAPI staining (blue). Scale bar, 50 μm. **(D)** Quantification of TH-positive neurite elongation in control (left panel) and *LRRK2* G2019S (right panel) iPSC-derived neurons (mean ± SEM, one-way ANOVA, Bonferroni post hoc, IFN-γ-treated vs untreated ****P*=0.0001, ***P*=0.0052; LRRK2 kinase inhibitor-treated vs untreated §§§§*P*<0.0001, §§§*P*=0.0004, 0.0003 in sequence, §§*P*=0.0044, 0.0068 in sequence; n=5 independent experiments).

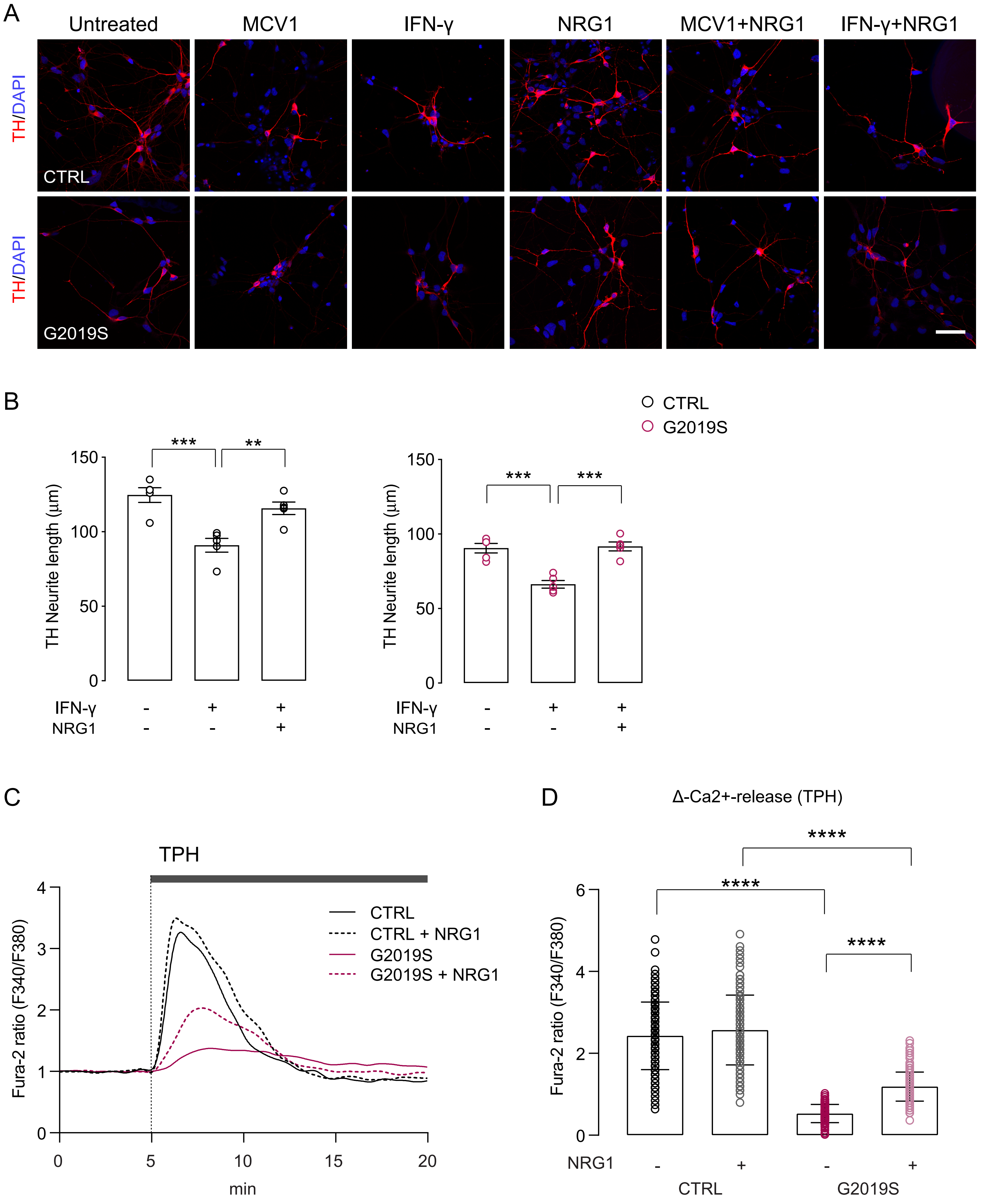

A
Untreated
MCV1
IFN-γ
NRG1
MCV1+NRG1
IFN-γ+NRG1
TH/DAPI
CTRL
G2019S
B
CTRL
G2019S
TH Neurite length (μm)
IFN-γ
NRG1
C
TPH
Fura-2 ratio (F340/F380)
min
CTRL
CTRL + NRG1
G2019S
G2019S + NRG1
D
Δ-Ca2+-release (TPH)
NRG1
CTRL
G2019S

Supplementary Figure 5. NRG1 rescues the impaired Ca^{2+}-storage capacity and neurite length defects induced by the *LRRK2* G2019S mutation and IFN-γ treatment. (A) Representative images of TH immunostaining (red) on *LRRK2* G2019S and isogenic control iPSC-derived neurons treated with 200IU/mL IFN-γ for 24 hrs with or without prior treatment with 200ng/mL NRG1 for 24 hrs. DAPI staining (blue). Scale bar, 50 μm. **(B)** Quantification of neurite elongation in control (left panel) and *LRRK2* G2019S (right panel) iPSC-derived neurons (mean ± SEM, one-way ANOVA, Bonferroni post hoc, ****P*=0.0007, 0.0002, 0.0001 in sequence, ***P*=0.0074; n=5 independent experiments). **(C)** Individual Ca^{2+} signaling traces of representative isogenic *LRRK2* G2019S and control neurons pretreated with 200 ng/mL NRG1 for 24 h or left untreated. **(D)** Quantification of TPH-mediated Δ-Ca2+ release is shown (mean ± SEM, two-way ANOVA, Bonferroni post hoc, *****P*<0.0001; 171 control, 149 NRG1-treated control, 90 G2019S and 200 NRG1-treated G2019S individual cells examined over 3 independent experiments).

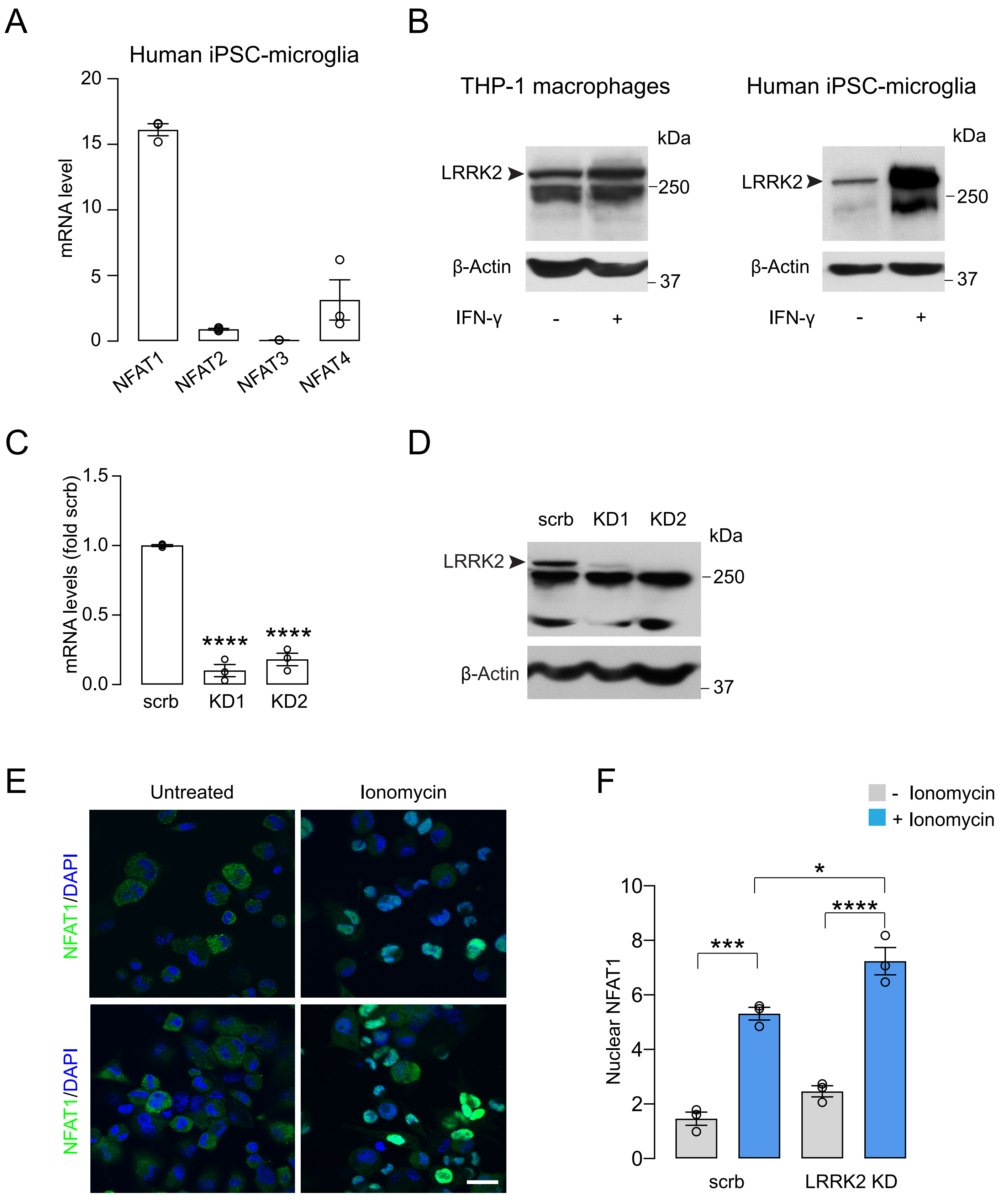
A
Human iPSC-microglia
mRNA level
NFAT1
NFAT2
NFAT3
NFAT4
B
THP-1 macrophages
Human iPSC-microglia
kDa
LRRK2
250
β-Actin
37
IFN-γ
C
mRNA levels (fold scrb)
****
scrb
KD1
KD2
D
scrb
KD1
KD2
kDa
LRRK2
250
β-Actin
37
E
Untreated
Ionomycin
NFAT1/DAPI
F
- Ionomycin
+ Ionomycin
Nuclear NFAT1
*
***
****
scrb
LRRK2 KD

Supplementary Figure 6. LRRK2 negatively regulates NFAT in human macrophages. (A) mRNA expression of NFAT isoforms in control iPSC-derived microglia measured by qRT-PCR (mean ± SEM; n=3 independent experiments). **(B)** THP-1 cells were differentiated into macrophages with 25 ng/mL phorbol-12-myristate-13-acetate (PMA) for 48 hrs. THP-1 macrophages (left panel) and human iPSC-derived microglia (right panel) were treated with 200 IU/mL IFN-γ for 72 hrs or left untreated. LRRK2 levels were assessed by Western blot (n=3 independent experiments). **(C)** Knockdown (KD) efficiency of two different *LRRK2*-targeting shRNAs (KD1 and KD2) in THP-1 cells determined by qRT-PCR, expressed as fold change over scramble (scrb) non-targeting shRNA (mean ± SEM, two-tailed t-test, ****P<0.0001; n=3 independent experiments). **(D)** Representative Western blot of LRRK2 showing knockdown efficiency in THP-1 macrophages. **(E)** Immunostaining for NFAT1 in scrb and *LRRK2* KD THP-1 macrophages. Cells were stimulated with 1 μM Ionomycin for 30 min or left untreated. NFAT1 (green), DAPI (blue). Scale bar, 20 μm. **(F)** Quantification of nuclear NFAT1 fluorescent intensity (mean ± SEM, two-way Anova with Bonferroni post hoc, ****P<0.0001, ***P=0.0002, *P=0.0166; n=3 independent experiments).

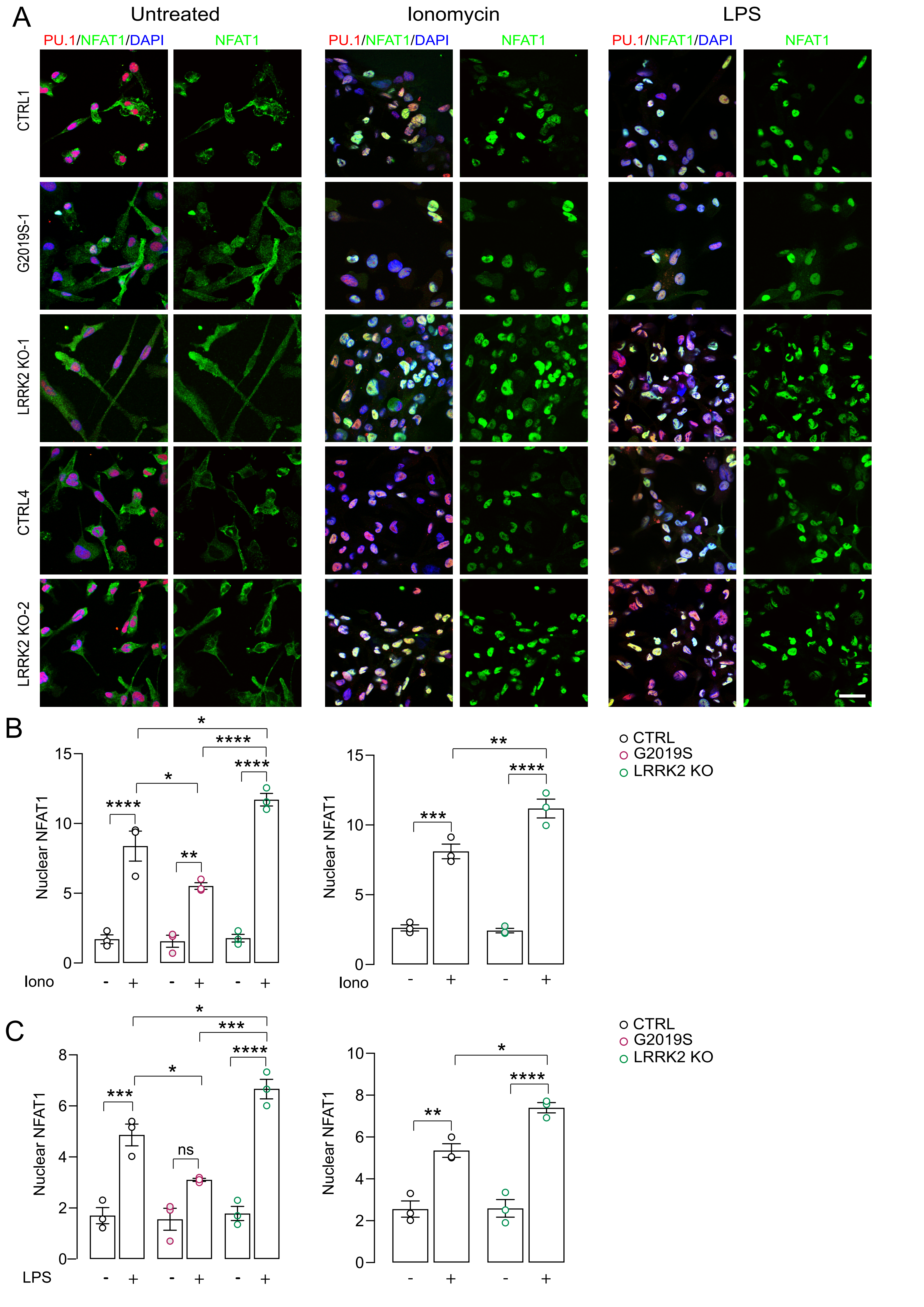
A
Untreated
Ionomycin
LPS
PU.1/NFAT1/DAPI
NFAT1
CTRL1
G2019S-1
LRRK2 KO-1
CTRL4
LRRK2 KO-2
B
Nuclear NFAT1
Iono
CTRL
G2019S
LRRK2 KO
C
LPS
ns

Supplementary Figure 7. LRRK2 negatively regulates NFAT in human iPSC-derived microglia. **(A)** Immunostaining for NFAT1 in isogenic CTRL, G2019S, and *LRRK2* KO iPSC-derived microglia. Cells were stimulated with 500 nM ionomycin or 100 ng/mL LPS for 30 min or left untreated. PU-1 (red), NFAT1 (green) and DAPI (blue) staining is shown. Scale bar, 20 μm. **(B, C)** Quantification of nuclear NFAT1 fluorescent intensity upon Ionomycin (B) or LPS (C) treatment in CTRL, *LRRK2* G2019S, and *LRRK2* KO iPSC-derived microglia (left panels) and a second CTRL and *LRRK2* KO isogenic couple (right panels) (mean ± SEM, two-way ANOVA, Bonferroni post hoc, *****P*<0.0001, ****P*=0.0001, 0.0004, 0.0001 in sequence, ***P*=0.0038, 0.0075, 0.0029 in sequence, **P*=0.0449, 0.0152, 0.0485, 0.0412, 0.0197 in sequence; n=3 independent experiments).

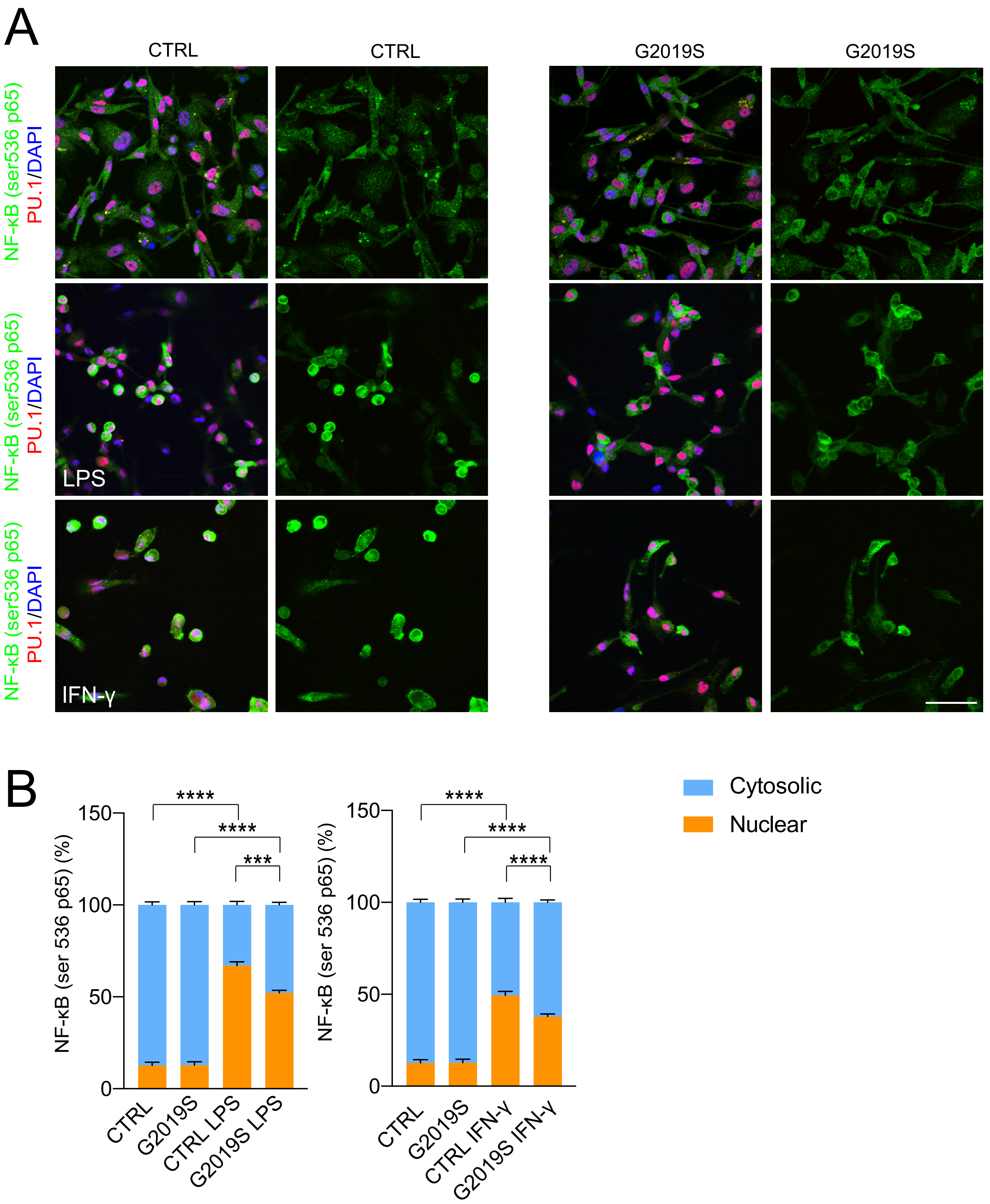
A
CTRL
CTRL
G2019S
G2019S
NF-κB (ser536 p65) PU.1/DAPI
NF-κB (ser536 p65) PU.1/DAPI
LPS
NF-κB (ser536 p65) PU.1/DAPI
IFN-γ
B
NF-κB (ser 536 p65) (%)
150
100
50
0
****
****
***
CTRL
G2019S
CTRL LPS
G2019S LPS
NF-κB (ser 536 p65) (%)
150
100
50
0
****
****
****
CTRL
G2019S
CTRL IFN-γ
G2019S IFN-γ
Cytosolic
Nuclear

Supplementary Figure 8. NF-κB p65 nuclear shuttling is impaired in *LRRK2* G2019S microglia. (A) Immunostaining for phospho-NF-κB p65 (Ser536) in isogenic CTRL and G2019S iPSC-derived microglia. Cells were treated with 100 ng/mL LPS for 24 hrs, 200 IU/mL IFN-γ for 24 hrs or left untreated. PU-1 (red), phospho-NF-κB (green) and DAPI (blue) staining is shown. Scale bar, 50 μm. **(B)** Quantification of nuclear and cytosolic phospho-NF-κB staining in CTRL and G2019S iPSC-derived microglia with or without LPS (left panel) or IFN-γ (right panel) treatment (mean ± SEM, two-way ANOVA, Bonferroni post hoc, ****P<0.0001, ***P=0.001; n=5 independent experiments).

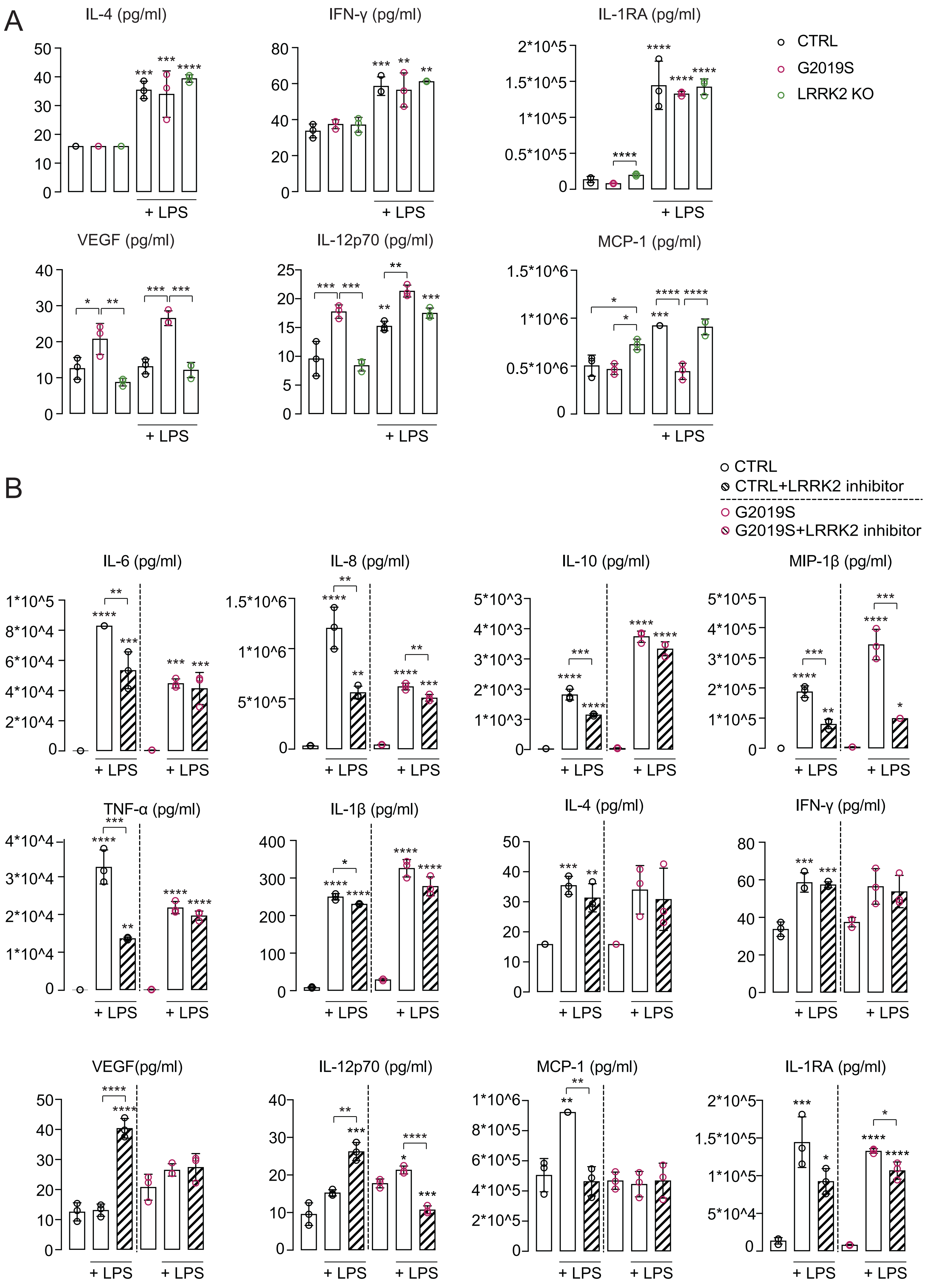
A
IL-4 (pg/ml)
IFN-γ (pg/ml)
IL-1RA (pg/ml)
CTRL
G2019S
LRRK2 KO
+ LPS
VEGF (pg/ml)
IL-12p70 (pg/ml)
MCP-1 (pg/ml)
B
CTRL
CTRL+LRRK2 inhibitor
G2019S
G2019S+LRRK2 inhibitor
IL-6 (pg/ml)
IL-8 (pg/ml)
IL-10 (pg/ml)
MIP-1β (pg/ml)
TNF-α (pg/ml)
IL-1β (pg/ml)
IL-4 (pg/ml)
IFN-γ (pg/ml)
VEGF(pg/ml)
IL-12p70 (pg/ml)
MCP-1 (pg/ml)
IL-1RA (pg/ml)
+ LPS

Supplementary Figure 9. LRRK2 regulates cytokine production in human iPSC-derived microglia. (A) CTRL, *LRRK2* G2019S, and *LRRK2* KO iPSC-derived microglia were treated with 100 ng/mL LPS for 24 hrs or left untreated, and cytokine levels were measured by Multiplex Elisa. Concentration of individual cytokines is shown (mean ± SEM, two-way ANOVA, Bonferroni post hoc, ****P<0.0001, ***P<0.001, **P<0.01, *P<0.05; n=3 independent experiments). **(B)** CTRL and G2019S iPSC-derived microglia were treated with 100ng/mL LPS for 24hrs with or without prior LRRK2 kinase inhibition with 100nM Mli-2. Concentration of individual cytokines, measured by Multiplex Elisa, is shown (mean ± SEM, one-way ANOVA, Bonferroni post hoc, ****P<0.0001, ***P<0.001, **P<0.01, *P<0.05; n=3 independent experiments).

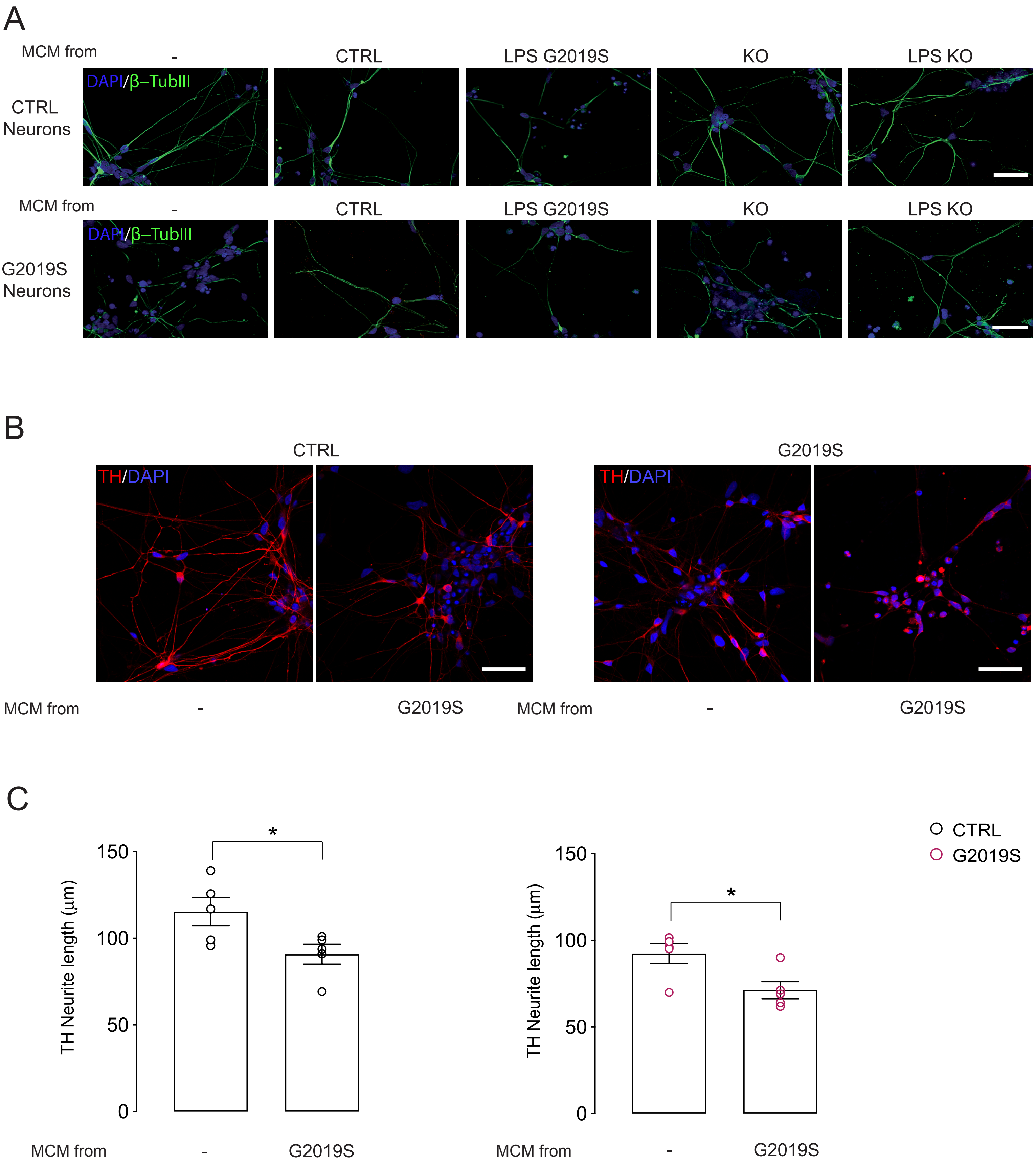

A
MCM from
-
CTRL
LPS G2019S
KO
LPS KO
CTRL Neurons
DAPI/β–TubIII
MCM from
-
CTRL
LPS G2019S
KO
LPS KO
G2019S Neurons
DAPI/β–TubIII
B
CTRL
G2019S
TH/DAPI
TH/DAPI
MCM from
-
G2019S
MCM from
-
G2019S
C
CTRL
G2019S
TH Neurite length (µm)
150
100
50
0
*
MCM from
-
G2019S
TH Neurite length (µm)
150
100
50
0
*
MCM from
-
G2019S

Supplementary Figure 10. *LRRK2* G2019S microglia activation induces neurite shortening in conditioned-medium experiments. CTRL and G2019S iPSC-derived neurons were exposed to isogenic CTRL, G2019S, or *LRRK2* KO LPS-activated microglial-conditioned media (MCM). **(A)** Representative images of β-TubIII immunostaining (green) showing neurite elongation in *LRRK2* G2019S and isogenic control iPSC-derived neurons stimulated with MCM. DAPI staining (blue). Scale bars, 50 μm. **(B)** Representative images of TH immunostaining (red) showing neurite elongation in *LRRK2* G2019S and isogenic control iPSC-derived neurons stimulated with MCM. DAPI staining (blue). Scale bars, 50 μm. **(C)** Quantification of TH-positive neurite elongation in control (left panel) and *LRRK2* G2019S (right panel) iPSC-derived neurons as shown on B (mean ± SEM, two-tailed t-test, *P=0.0391, 0.0237 in sequence; n=5 independent experiments).
