# Supplementary material for: Interferon-γ signaling synergizes with LRRK2 in neurons and microglia derived from human induced pluripotent stem cells
Source: Nat Commun. 2020 Oct 14;11:5163. doi: 10.1038/s41467-020-18755-4 (PMC7560616; doi:10.1038/s41467-020-18755-4)
Supplement: Supplementary file 3 — Reporting Summary [file 41467_2020_18755_MOESM3_ESM.pdf]

nature research

Corresponding author(s): Michela Deleidi

Last updated by author(s): 2020/09/06

Reporting Summary

Nature Research wishes to improve the reproducibility of the work that we publish. This form provides structure for consistency and transparency in reporting. For further information on Nature Research policies, see Authors & Referees and the Editorial Policy Checklist.

Statistics

For all statistical analyses, confirm that the following items are present in the figure legend, table legend, main text, or Methods section.

n/a	Confirmed	
☐	☒	The exact sample size (n) for each experimental group/condition, given as a discrete number and unit of measurement
☐	☒	A statement on whether measurements were taken from distinct samples or whether the same sample was measured repeatedly
☐	☒	The statistical test(s) used AND whether they are one- or two-sided<br>*Only common tests should be described solely by name; describe more complex techniques in the Methods section.*
☒	☐	A description of all covariates tested
☐	☒	A description of any assumptions or corrections, such as tests of normality and adjustment for multiple comparisons
☐	☒	A full description of the statistical parameters including central tendency (e.g. means) or other basic estimates (e.g. regression coefficient) AND variation (e.g. standard deviation) or associated estimates of uncertainty (e.g. confidence intervals)
☐	☒	For null hypothesis testing, the test statistic (e.g. F, t, r) with confidence intervals, effect sizes, degrees of freedom and P value noted<br>*Give P values as exact values whenever suitable.*
☒	☐	For Bayesian analysis, information on the choice of priors and Markov chain Monte Carlo settings
☒	☐	For hierarchical and complex designs, identification of the appropriate level for tests and full reporting of outcomes
☒	☐	Estimates of effect sizes (e.g. Cohen's d, Pearson's r), indicating how they were calculated

Our web collection on statistics for biologists contains articles on many of the points above.

Software and code

Policy information about availability of computer code

Data collection	Images were acquired with a Leica CM 1900 instrument or Zeiss Imager Z1 with Apotome (Carl Zeiss) microscope. Calcium imaging was performed with an upright fluorescence microscope (BX50WI, Olympus). Multiplex Elisa was performed on a FLEXMAP 3D® analyzer system. Extracellular acidification rate was analyzed using an XFe96 Extracellular Flux Analyzer (Seahorse Biosciences).
Data analysis	Data were analyzed using GraphPad Prism 8.00 software and MATLAB R2019B.<br>Image J was used for image analysis and quantification. dSTORM images were further analyzed with the Matlab-based open source software SMLM image filament network extractor (SIFNE), keeping the parameters constant among the experiments 42. Otsu´s threshold was used to extract the binarized image. Filaments shorter than 0.2 µm were excluded from analysis.<br>Miltiplex Elisa data were analyzed using xPONENT® 4.2 software (Luminex, Austin, TX, USA).<br>Statistical testing involved two-tailed Student's t-test, multiple t-test, one-way ANOVA, two-way ANOVA, followed by Bonferroni multiple comparison test or Kolmogorov–Smirnov test, as indicated. Data are expressed as mean ± SEM. The significance level was set at P<0.05.

For manuscripts utilizing custom algorithms or software that are central to the research but not yet described in published literature, software must be made available to editors/reviewers. We strongly encourage code deposition in a community repository (e.g. GitHub). See the Nature Research guidelines for submitting code & software for further information.

Data

Policy information about availability of data

All manuscripts must include a data availability statement. This statement should provide the following information, where applicable:

- Accession codes, unique identifiers, or web links for publicly available datasets
- A list of figures that have associated raw data
- A description of any restrictions on data availability

Source data are provided with this paper.

Field-specific reporting

Please select the one below that is the best fit for your research. If you are not sure, read the appropriate sections before making your selection.

☒ Life sciences ☐ Behavioural & social sciences ☐ Ecological, evolutionary & environmental sciences

For a reference copy of the document with all sections, see nature.com/documents/nr-reporting-summary-flat.pdf

Life sciences study design

All studies must disclose on these points even when the disclosure is negative.

Sample size	Sample size was chosen based on previous experience and standards in the field.
Data exclusions	No data were excluded from the analyses.
Replication	All attempts at replication were successful.
Randomization	Cells were randomly distributed to cell-culture multi-well plate positions and randomly assigned to experimental groups. No additional randomization was applicable to the current study. Cells were grouped based on the genotype/treatment and compared with the respective control.
Blinding	Investigators were blinded to the groups and samples.

Reporting for specific materials, systems and methods

We require information from authors about some types of materials, experimental systems and methods used in many studies. Here, indicate whether each material, system or method listed is relevant to your study. If you are not sure if a list item applies to your research, read the appropriate section before selecting a response.

Materials & experimental systems

n/a	Involved in the study
☐	☒ Antibodies
☐	☒ Eukaryotic cell lines
☒	☐ Palaeontology
☒	☐ Animals and other organisms
☒	☐ Human research participants
☒	☐ Clinical data

Methods

n/a	Involved in the study
☒	☐ ChIP-seq
☒	☐ Flow cytometry
☒	☐ MRI-based neuroimaging

Antibodies

Antibodies used

Western Blot primary antibodies:
rat monoclonal anti-LRRK2 24D8 (kind gift from C. Johannes Gloeckner; 2 ug; 100ug/ml, 1:50);
rat monoclonal anti-LRRK2 1E11 (kind gift from C. Johannes Gloeckner; 4 ug; 100ug/ml, 1:25);
rabbit monoclonal anti LRRK2 [MJFF2 (c41-2)] (1:1000, Abcam, Cat. No. ab133474);
rabbit anti-LRRK2 (UDD3 30[12])(1:500; Abcam, Cat. No. ab133518);
rabbit anti-AKT3 (1:1000, Cell Signaling Technology, Cat. No. 4059);
rabbit anti-NFAT3 (23E6) (1:1000, Cell Signaling Technology, Cat. No. 2183);
rabbit anti-PARP (1:8000, Cell Signaling Technology, Cat. No. 9542);
rabbit anti HSP90 (1:15000, Enzo, Cat. No. ADI-SPA-836F);
mouse anti-β-Actin (1:20000, Sigma, Cat. No. A5441);
rabbit anti-AKT (1:1000, Cell Signaling Technology, Cat. No.9272);
rabbit anti-phospho-AKT (Ser473) (1:1000, Cell Signaling Technology, Cat. No.4060)

Western Blot secondary antibodies:
anti-rabbit IgG, HRP-linked (1:10.000, Cell Signaling Technology, Cat. No.7074);
anti-mouse IgG, HRP-linked (1:10.000, Cell Signaling Technology, Cat. No.7076);
anti-rat; IgG, HRP-linked (1:10.000, Cell Signaling Technology, Cat. No.7077)

Immunocytochemistry primary antibodies:
rabbit anti NFAT1 (D43B1) (1:1000, Cell Signaling Technology, Cat. No. 5861);
rabbit anti NFAT3 (phospho-S676) (1:250, Biorbyt, Cat. No. orb256717);
mouse anti-Tubulin β3 (TUBB3) (1:1000, Previously Covance, Cat. No. MMS-435P);
rabbit anti-Tyrosine Hydroxylase (1:500, Pel-Freez, Cat. No. P40101-150);
mouse anti Spi1/PU.1 (1:100, Biolegend, Cat. No. 658002);

rabbit anti-Iba1 (1:2000, Wako Chemicals, Cat. No. 016-20001);
rabbit anti-phospho-NFκB p65 (Ser536; 1:500; Thermo Fisher, Cat. No. MA5-15160).

Immunocytochemistry secondary antibodies:
Goat anti-mouse IgG (H+L), Alexa Fluor488 (1:1000, Invitrogen, Cat. No. A-11001);
Goat anti-rabbit IgG (H+L), Alexa Fluor488 (1:1000, Invitrogen, Cat. No. A-11008);
Goat anti-mouse IgG (H+L), Alexa Fluor568 (1:1000, Invitrogen, Cat. No. A-11004);
Goat anti-rabbit IgG (H+L), Alexa Fluor568 (1:1000, Invitrogen, Cat. No. A-11011);
Goat anti-mouse IgG (H+L), Alexa Fluor647 (1:1000, Invitrogen, Cat. No. A-21236)

Validation

All the antibodies used in the study are common, well- established commercial antibodies. Validation data are shown for each antibody by the manufacturers.

Eukaryotic cell lines

Policy information about cell lines

Cell line source(s)

All iPSCs used in the study were derived from patients who signed an informed consent . The Ethics Committee of the Medical Faculty and the University Hospital Tübingen (Ethikkommission der Medizinischen Fakultät am Universitätsklinikum Tübingen) had approved the protocol before performing the experiments.

NFAT Reporter - HEK293 Cell Line (PKC/ Ca2+ Pathway) cell lines were purchased from BPS Bioscience

THP-1 cell lines were purchased from Sigma Aldrich.

Authentication

The identity of iPSC cell lines was routinely confirmed by Sanger sequencing.

Mycoplasma contamination

All cell lines were routinely assessed for mycoplasma using a Venor®GeM Classic kit (Minerva Biolabs) and tested negative.

Commonly misidentified lines
(See ICLAC register)

There are no commonly misidentified cell lines.
